# Current Animal Models of Postoperative Spine Infection and Potential Future Advances

**DOI:** 10.3389/fmed.2015.00034

**Published:** 2015-05-26

**Authors:** A. I. Stavrakis, A. H. Loftin, E. L. Lord, Y. Hu, J. E. Manegold, E. M. Dworsky, A. A. Scaduto, N. M. Bernthal

**Affiliations:** ^1^Department of Orthopaedic Surgery, Center for Health Sciences, Orthopaedic Hospital Research Center, David Geffen School of Medicine, University of California Los Angeles, Los Angeles, CA, USA

**Keywords:** animal, model, spine, postoperative infection, review

## Abstract

Implant related infection following spine surgery is a devastating complication for patients and can potentially lead to significant neurological compromise, disability, morbidity, and even mortality. This paper provides an overview of the existing animal models of postoperative spine infection and highlights the strengths and weaknesses of each model. In addition, there is discussion regarding potential modifications to these animal models to better evaluate preventative and treatment strategies for this challenging complication. Current models are effective in simulating surgical procedures but fail to evaluate infection longitudinally using multiple techniques. Potential future modifications to these models include using advanced imaging technologies to evaluate infection, use of bioluminescent bacterial species, and testing of novel treatment strategies against multiple bacterial strains. There is potential to establish a postoperative spine infection model using smaller animals, such as mice, as these would be a more cost-effective screening tool for potential therapeutic interventions.

## Introduction

Postoperative infection is a devastating complication following implant related spine surgery and can lead to neurological compromise, disability, and even increased morbidity and/or mortality. In comparison to other types of orthopedic infections, explantation of hardware is avoided in postoperative spine infections as this would render the spine unstable and could potentially lead to neurologic compromise. This complication also has a detrimental effect on the healthcare system, with patients requiring several hospitalizations, repeat surgeries, and a long course of intravenous followed by oral antibiotics. This amounts to huge costs, with the treatment of a single implant-associated spinal wound infection potentially costing more than $900,000 (1). Despite advances in aseptic surgical technique and perioperative antibiotic use, postoperative infection still occurs in approximately 1% of elective spine surgery without the use of hardware and 3.4–8.5% when hardware is used (2–6). This rate surpasses 10% with certain patient and operative risk factors. Diabetes, obesity, immunocompromised state, advanced age, trauma, and certain pediatric disorders (i.e., neuromuscular scoliosis) have been well documented as risk factors for increased infection following spine surgery (7). Multilevel or revision surgery, use of instrumentation, and significant intraoperative blood loss also greatly increase the risk of infection (2, 8, 9). Stainless steel spinal implants are also associated with higher rates of infection when compared to titanium and chromium-cobalt constructs (10). Postoperative infection additionally increases the risk of pseudoarthrosis, with an increased risk from 11.5% to 29.7% when infection is present (11).

Treatment of postoperative spine infections becomes extremely challenging. The most common organism isolated from implant related spine infections is *Staphylococcus* species, with *Staphylococcus aureus* (*S. aureus*) being the most common and *Staphylococcus epidermidis* being the second most common (9). *Propionibacterium acnes* (*P. acnes*), a normal inhabitant of skin flora, is also a common pathogen in implant related spine surgery (12). Bacteria readily adhere to the foreign implant surface. Over the course of several days, bacteria produce a polysaccharide (glycocalyx) biofilm layer which covers the implant surface, preventing antibiotic and immune cell penetration. Once a biofilm is established, bacteria become 100–1,000 times less susceptible to antibiotics (9).

Given the high rate of infection following spine surgery and the significant morbidity associated with this complication, further basic science and clinical research are needed to better understand the pathogenesis of this devastating complication. As in many areas of medicine, animal models provide a way to better understand the pathophysiology of this disease process and to evaluate potential treatment options. This paper provides an overview of the existing animal models of postoperative spine infection and highlights the strengths and weaknesses of each model. In addition there is discussion regarding potential modifications to these animal models to better evaluate preventative and treatment strategies for this challenging complication.

## Established Animal Models of Spine Infection

### Initial animal model of postoperative spine infection

In 1998, Guiboux et al. established the first spine infection animal model. Guiboux et al. combined previously described rabbit spine fusion, instrumentation, and intervertebral disk infection models to create a postoperative infection model (13). Twenty rabbits were inoculated with *S. aureus* intraoperatively and were split into four groups based on whether or not they received instrumentation and prophylactic perioperative antibiotics (cefazolin 30 mg/kg 5 min before incision or after surgery).

A skin incision was made just posterior to the L4 and L5 posterior spinous processes. The lumbodorsal fascia over these spinous processes was then split longitudinally. The paravertebral muscles were elevated from the underlying bone, exposing the lamina and facet joints. The posterior spinous processes of L4 and L5 were then removed. The lamina and pars intraarticularis were then decorticated.

For the animals receiving instrumentation, a 26-gage wire was double-braided and placed around the L3/L4 and L4/L5 facet joints bilaterally in a figure of eight configuration. Autogenous bone graft was then placed on the decorticated fusion bed in all animals. Following bone grafting, 1 × 10^3^ colony-forming units (CFU) *S. aureus* in a 0.05 ml saline solution was applied onto the bone grafted and hardware (if applicable) region. The surgical site was then closed in layers. On postoperative day (POD) 5, the animals were euthanized and the surgical sites opened. Swab and tissue cultures were obtained from all the animals and used to evaluate postoperative infection.

In this study, based on surgical site aerobic swabs and tissue cultures obtained on POD 5, all the animals who did not receive any prophylactic antibiotics developed infection and all the animals who received preoperative or postoperative cefazolin had negative *S. aureus* cultures, regardless of whether or not hardware was implanted.

### Rat pedicle screw model

**Figure 1:**
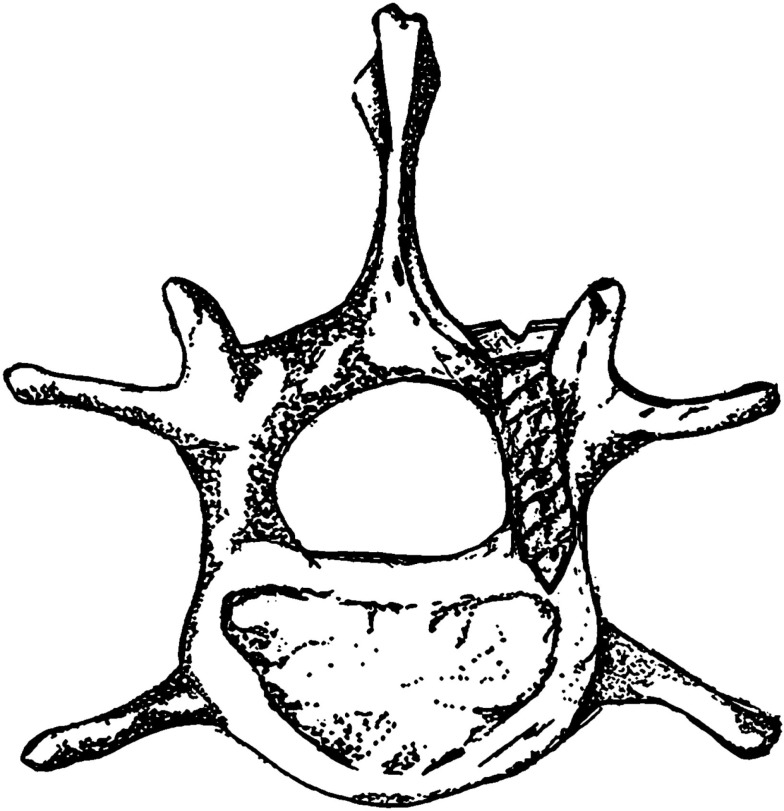
**Rat thoracic vertebra with pedicle screw**.

A rat model of pedicle screw *S. aureus* infection, which included a sample size of 40 animals, was published by Ofluoglu et al. in the Archives of Orthopaedic and Trauma Surgery in 2007 (14). Under sterile conditions, a 1.5 cm longitudinal midline skin incision was made in the midline thoracolumbar area (T10–L1) with a scalpel. The paravertebral muscles were separated from the spinous processes and laminas and the facet joints were exposed. The lamina was decorticated by a scalpel and a 20-gage needle was used to ream an opening through the junction of the lamina and facet joint. A 1-mm diameter and 3-mm long titanium screw was then inserted from the lamina into the pedicle. A 10 μl solution of sterile saline or *S. aureus* (1 × 10^2^ CFU, 1 × 10^3 ^CFU, or 1 × 10^6 ^CFU) was then placed onto the screw head and surrounding tissue and the tissue was then sutured closed. All animals were euthanized on POD 15. Figure 1 shows a representative image of the positioning of the pedicle screw in the vertebra.

No animals had any obvious neurologic deficits postoperatively. On POD 15, animals underwent microbiologic or histopathologic evaluation of the implant and bone/soft tissue surrounding the implant. All rats in the *S. aureus* group developed evidence of osteomyelitis postoperatively based on histology studies. Bacteria were present on all implants except for one. No rats in any of the inoculum groups had signs of bacteremia/sepsis (as evidenced by negative blood cultures, normal vital signs postoperatively). Interestingly, only the 1 × 10^6^ CFU *S. aureus* group had histopathologic evaluation of acute osteomyelitis, as evidenced by osteonecrosis and neutrophil leukocyte infiltration. Given these findings, these authors conclude that the optimal inoculum of *S. aureus* in a rat model of spine infection is 1 × 10^6^ CFU.

## Rabbit partial laminectomy model

**Figure 2:**
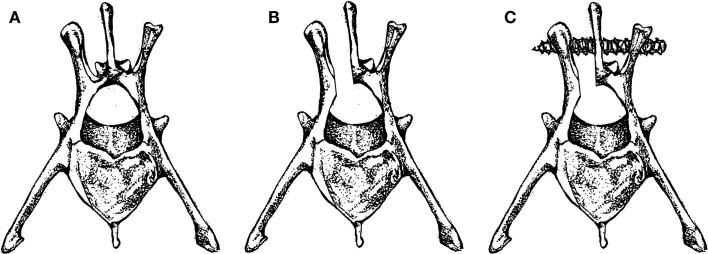
**(A)** Rabbit vertebra with **(B)** partial laminectomy and **(C)** screw fixation.

In 2000, Poelstra et al. published a rabbit spinal implant model of methicillin-resistant *S. aureus* (MRSA) in multiple non-contiguous surgical sites in the lumbar region (15). Eight rabbits were included in this study. Under sterile conditions, a 2.5 cm dorsal skin incision was made longitudinally in the midline, followed by a single incision in the fascia to expose the spinous process which was then excised using a small rongeur to mimic a partial laminectomy (Figures 2A,B). This was then followed by implantation of a 0.85 mm diameter stainless steel threaded Kirschner wire (K-wire) implantation into the transverse processes of T13, L3, and L6 vertebrae. T13 and L6 levels were inoculated with a 100 μl solution of sterile saline or varying amounts of MRSA (1 × 10^2^, 1 × 10^3^, 1 × 10^4^, or 1 × 10^5 ^CFU), the L3 level was used as a sterile control (Figure 2C). The fascia and skin were then sutured closed. This was then repeated for the remaining two levels through a separate incision.

The animals were euthanized on POD 7 and biopsies were performed to evaluate for implant related infection. Based on biopsy cultures, all the sites inoculated with a minimum of 1 × 10^3^ CFU MRSA developed infection. An inoculum of 1 × 10^2^ CFU, however, did not consistently result in infection by POD 7. The authors thus recommend an inoculum in this model of at least 1 × 10^3^ CFU. On POD 7, there were no signs of bacteremia as evidenced by negative blood cultures in all animals. Additionally, none of the L3 level control sites between the infected vertebrae developed infection, making this level a good control.

Several other groups use this animal model to evaluate the efficacy of various infection prevention strategies. One such study evaluated the ability of local vancomycin powder in eradicating surgical site infection following spine implantation surgery (16). Rabbits received either preoperative cefazolin (30 mg/kg administered 15 min prior to incision) or preoperative cefazolin of the same dose plus intraoperative vancomycin powder. The experimental group was treated with 100 mg of vancomycin powder (equivalent to 2 g dose in an 80 kg human) placed directly within the wound prior to closure. On POD 4 the rabbits were euthanized and tissue samples were collected for culture. The implants were also retrieved and cultured. All vancomycin-treated rabbits had negative cultures and all animals in the control group had positive cultures. The authors conclude that intraoperative vancomycin powder helps reduce the risk of surgical site infections.

Another study used this animal model to evaluate the ability of controlled release gentamicin via polylacticoglycolic acid (PLGA) linked microspheres, with a 3–7 day resorption, in preventing *S. aureus* infection. All animals were given intravenous ceftriaxone (20 mg/kg) prior to incision. The surgical technique described above was then performed. Following implant placement, 1 × 10^6^ CFU *S. aureus* was then inoculated. The animals were then treated with gentamicin PLGA microspheres (treatment group) or PLGA carrier (control group). Animals were euthanized on POD 7, implant and soft tissue cultures were then obtained using the same method described above. Using this model, the researchers found that postoperative infection on POD 7 was reduced by at least 50% using intraoperative gentamicin microspheres (1).

## Discussion

In addition to being reproducible, the animal models described are effective in their simulation of surgical technique (Table 1). Guiboux et al. described rabbit spine fusion, instrumentation, and intervertebral disk infection models. Unfortunately, results from therapeutic evaluation studies were not consistent with those in real patients as a significant number of patients developed postoperative infection regardless of the administration of preoperative antibiotics. The discrepancy between the findings in this model and in real patients perhaps can be attributed to the small sample size and presumably by low sensitivity of the culture techniques used in this study. Sensitivity in this study may have been improved if the implants had been extracted and cultured separately in addition to surgical site aerobic swabs and tissue cultures. Some potential modifications to this model include evaluation of infection at multiple time points as well as quantification of infection severity.

**Table 1:** **Summary of main models of postoperative spine infection**.

Authors	Descriptions of model	Evaluation technique	Advantage/uses	Disadvantages/limitations
• Guiboux et al.	• Postoperative infection rabbit model with implantation in L4/L5 facet joint. Used to assess the efficacy of prophylactic therapeutic regimens of cefazolin in preventing iatrogenic *S. aurues* infections	• Surgical swabs taken on POD 5 and evaluated for infection	• Model is valid and reproducible	• No longitudinal evaluation
			• Model is accurate in simulating surgical technique	• Results are not consistent with patient results
				• Small sample size
				• Low sensitivity of evaluation techniques
• Ofluoglu et al.	• Spine infection rat model with implantation in the thoracolumbar area after laminar decortication. Used to evaluate the ideal inoclum of *S. aurues*	• Microbiological evaluation of microscrews, bone and tissue	• Surgical technique closely mimics implantation of pedicle screws	• No longitudinal evaluation • Biofilm formation was not evaluated
		• Histopathologic evaluation of the implant and bone/soft tissue	• Model is reproducible	
• Poelstra et al.	• Rabbit spinal model with implantation into transverse process. Infection of methicillin-resistant *S. aurues* (MRSA) was compared to non-contiguous surgical sites in the lumbar spine	• Tissue and implants were evaluated for colony-forming units	• Reproducible	• No longitudinal evaluation
			• Close simulation of human local surgical site (local soft tissue damage and increased dead space)	• Internal control may not be representative of unaffected area
			• Internal control in each animal that allows for effective comparisons of treatment strategies and biomaterials	

Ofluoglu et al. successfully mimicked the surgical technique of pedicle screw implantation, the most commonly used instrumentation in spine procedures. In addition to obtain cultures of the tissue and implant to detect signs of infection, the authors looked specifically at osteomyelitis via histologic analysis. However, histological signs of the osteomyelitis, as evidenced by osteonecrosis and neutrophil and leukocyte infiltration were only observed at 10^6^ CFU concentrations.

Poelstra et al. developed a unique study in which multiple implants and sites of infection can be evaluated in the same animal. This allows for an internal control in each animal and allows for more effective comparisons of treatment strategies and various implant materials. One should do so with caution, however, given that infection stresses the immune system, and thus an internal control such as this one which is surrounded both proximally and distally with infection is not a true control. The surgical technique in this study involves a partial laminectomy, which creates local soft tissue damage and increased dead space at the surgical site. This technique closely simulates the human local surgical site environment and an important topic of study as this region of dead space fills with hematoma, becoming a very favorable environment for bacteria to thrive.

The ideal spine infection animal model offers anatomic similarities to human anatomy and accurately evaluates infection longitudinally using multiple techniques. Examples of such techniques include bacterial burden quantification via culturing and imaging, scanning electron microscopy to evaluate for biofilm presence, and histologic analysis.

Future models would ideally be used to longitudinally track infection over several days or weeks, not just at a single time point, which requires animal euthanasia for any indication of bacterial burden. One plausible approach would be to integrate advanced imaging techniques that allow for assessment of bacterial burden at several time points in the same animal, such as through the use of bioluminescent bacterial strains.

Additionally, all current animal models have only evaluated postoperative infection using *S. aureus* as the pathogen. Ideally, future models would be able to evaluate infection with several different bacterial strains, such as *S. epidermidis* and *P. acnes*. This would be particularly useful in testing potential novel therapeutic strategies given that these other organisms are also responsible for a great percentage of such infections.

Lastly, there is potential to establish a postoperative spine infection model using smaller animals, such as mice, as these would be a more cost-effective screening tool for potential therapeutic interventions. If such therapies prove promising, they may then be tested in larger animal models and eventually in humans. Mice also offer the opportunity to study immunomodulation via the use of genetically modified animals. Immunomodulation may allow us to better understand the underlying pathophysiology and immune response of such infection.

## Conclusion

The established animal models described offer many strengths and weaknesses. Potential modifications to these models have been suggested so that they may better evaluate preventative and treatment strategies for implant related spine infection.

Guiboux et al., Ofluoglu et al., and Poelstra et al. were effective in designing models that are reproducible and accurately simulate surgical technique. In addition, Poelstra et al., was successful in establishing an internal control in his rabbit model. However, questions still remain on the sensitivity of assays performed in these studies, which are small volume studies and which only evaluate infection at one time point by relying on simple bacteria culture methods and histologic analysis.

There is potential for future models to address these limitations. Future models may track infection longitudinally for several days or weeks by possibly utilizing advanced imaging techniques that allow for assessment of bacterial burden *in vivo*. Furthermore, new models may utilize multiple bacterial strains and species including *S. epidermidis* and *P. acnes*. Bioluminescent bacterial strains may provide a means of visualizing infection. Lastly, there is potential to explore a postoperative spine infection model in more cost-effective animals such as mice, which also offer opportunities to study immunomodulation. The development of a model that successfully addresses these limitations would allow for assessment of potential future advances in the treatment of postoperative spine infection.

## Conflict of Interest Statement

N. M. Bernthal is a paid consultant of Plexxicon. The rest of the authors have nothing to disclose.
